# The application of mechanical load onto mouse tendons by magnetic restraining represses Mmp-3 expression

**DOI:** 10.1186/s13104-023-06413-z

**Published:** 2023-06-30

**Authors:** Rouhollah Mousavizadeh, Valerie C. West, Kameron L. Inguito, Dawn M. Elliott, Justin Parreno

**Affiliations:** 1 Department of Physical Therapy, Faculty of Medicine, The University of British Columbia, Vancouver, Canada; 2 Department of Biomedical Engineering, University of Delaware, Newark, DE USA; 3 Department of Biological Sciences, University of Delaware, Newark, DE USA

## Abstract

**Objectives:**

Mechanical loading is crucial for tendon matrix homeostasis. Under-stimulation of tendon tissue promotes matrix degradation and ultimately tendon failure. In this study, we examined the expression of tendon matrix molecules and matrix-degrading enzymes (matrix metalloproteinases) in stress-deprived tail tendons and compared to tendons that were mechanically loaded by a simple restraining method.

**Data description:**

Isolated mouse tail fascicles were either floated or restrained by magnets in cell culture media for 24 h. The gene expression of tendon matrix molecules and matrix metalloproteinases in the tendon fascicles of mouse tails were examined by real-time RT-PCR. Stress deprivation of tail tendons increase Mmp3 mRNA levels. Restraining tendons represses these increases in Mmp3. The gene expression response to restraining was specific to Mmp3 at 24 h as we did not observe mRNA level changes in other matrix related genes that we examined (Col1, Col3, Tnc, Acan, and Mmp13). To elucidate, the mechanisms that may regulate load transmission in tendon tissue, we examined filamentous (F-)actin staining and nuclear morphology. As compared to stress deprived tendons, restrained tendons had greater staining for F-actin. The nuclei of restrained tendons are smaller and more elongated. These results indicate that mechanical loading regulates specific gene expression potentially through F-actin regulation of nuclear morphology. A further understanding on the mechanisms involved in regulating Mmp3 gene expression may lead to new strategies to prevent tendon degeneration.

## Introduction

Mechanical stimuli is crucial for tendon homeostasis and repair [1, 2]. Mechanical loading on tendons can regulate both the synthesis and degradation of collagen. Thus, the strength of tendon tissue [3, 4] is determined by the amount of mechanical stimulation received by tenocytes [5]. Insufficient mechanical load throughout a tendon as a result of tendon detachment or rupture, can lead to cellular under stimulation and the induction of matrix metalloproteinases (MMPs) [6]. MMPs are a major contributor to tissue degeneration [7]. Therefore, the dysregulation of MMPs can affect long-term healing outcomes.

MMP3 is a critical MMP that may regulate tendon pathology. MMP3 can broadly degrade extracellular matrix proteins (i.e., fibronectin, laminin, proteoglycans, etc.) and can activate other MMPs, having the ability to increase MMP1 activity eightfold [8]. The elevated expression of MMP3 has been associated with both tendon tearing [9] as well as recurrence of postoperative tendon tears [10]. The Mmp3 gene has been shown to be mechanosensitive in a variety of cells. In addition to tendon cells, cartilage and bone-derived cells show changes in Mmp3 mRNA levels in response to mechanical load [11–17]. In tendons, a widely used method to study stress deprivation is by culturing tail tendon explants floating in tissue culture. Using stress deprivation cultures, we and others have shown a selective increase in Mmp3 expression in stress-deprived tendon tissue [17–20].

A better understanding of the mechanotransduction mechanisms that regulate Mmp3 may enable the generation of new therapeutic targets against tendon pathology. One prospective mechanism is that stress deprivation interferes with the transmission of mechanical loads onto the nucleus via the F-actin cytoskeleton [21–23], resulting in chromatin reorganization which alters gene expression [24, 25]. While the transmission of loads may be disrupted under stress deprivation, there is limited evidence for the regulation of F-actin by mechanical loads during tendon stress deprivation. Here, we hypothesized that mechanically loading tendons in culture prevents F-actin depolymerization, nuclear rounding, and represses Mmp3 gene expression. To test this hypothesis, we developed a simple, inexpensive model to prevent stress deprivation by restraining tail tendons under commercially available ceramic magnets.

## Materials and methods

### Tissue isolation and culture

Healthy female and male wild-type C57Bl6/J mice between the ages of 8–10 week were used in this study. Breeding pairs were originally purchased from The Jackson Laboratory (Bar Harbor, ME). All procedures were conducted following approved animal protocols from the University of Delaware Institutional Animal Care and Use Committee (IACUC). Following euthanasia by CO_2_ inhalation, tendon fascicles were isolated from the tails of wild-type C57BL/6J mice as previously described [26]. The fascicles were immediately washed in Dulbecco’s Modified Eagles’ Media (DMEM) consisting of 1% antimycotic/antibiotic. Fascicles were then randomized into two groups: stress deprived or restrained. The fascicles in the stress deprived group were suspended in a petri dish consisting of 25mL DMEM.

### Mechanical restraining of tendons

**Fig. 1:**
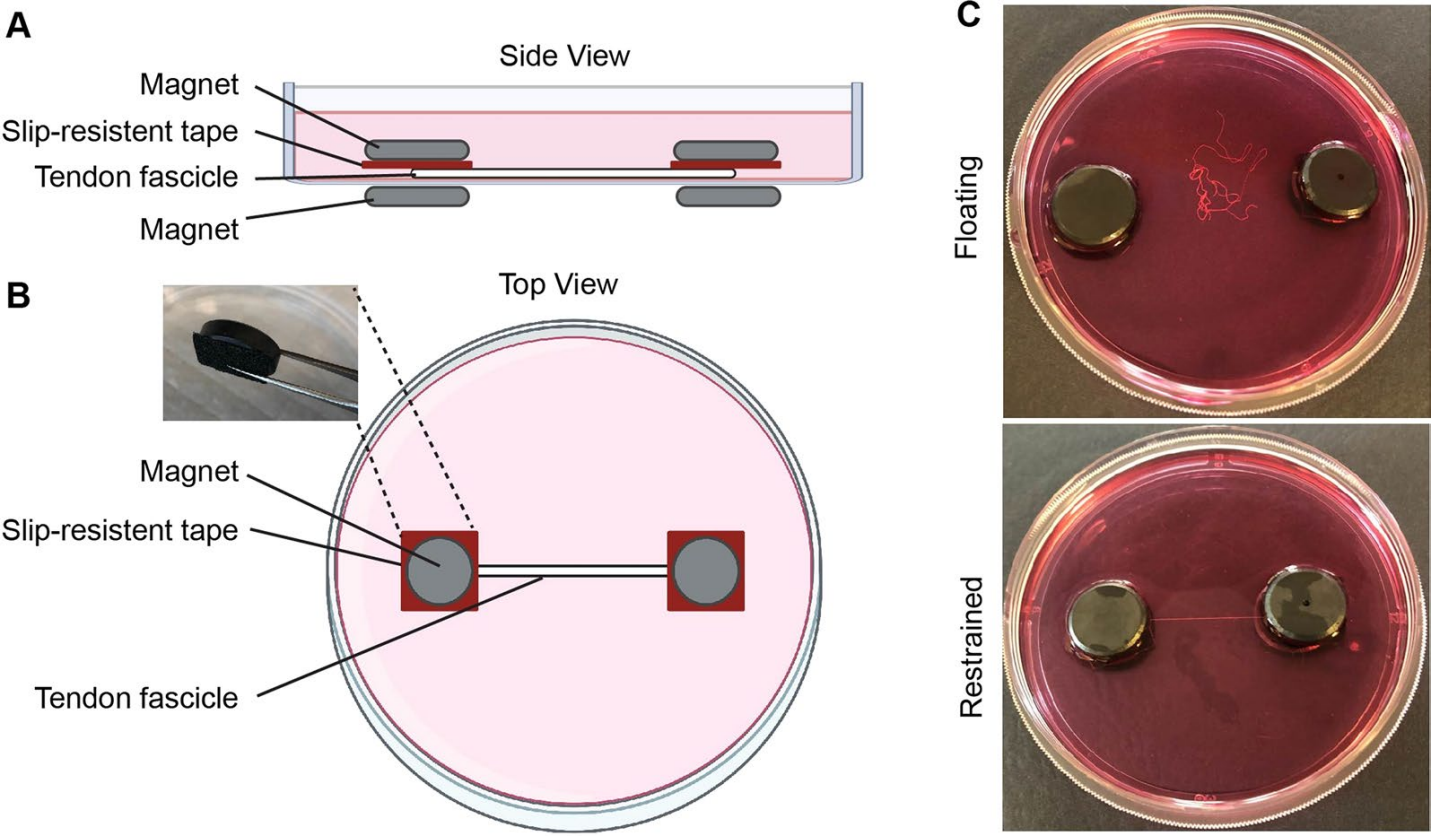
Simple, inexpensive method to mechanically load tendons. **A** Side and **B** top view schematics showing tendon fascicles were mechanically restrained under magnets. Inset in **B** shows magnet with attached slip resistant tape. **C** Image showing tendons that were stress deprived (Floating) versus those that were mechanically loaded (Restrained)

To mechanically restrain tendons, two 18 mm round ceramic magnets (Anpro; Amazon) were placed on the underside of a standard petri dish. During the restraining procedure a small amount of media (~ 1-2mL) were placed on the petri dish to avoid drying out of the fascicles. Individual fascicles were then carefully extended on the petri dish with one fascicle end over a magnet (magnet 1, Fig. 1). A second magnet (magnet 2) with pre-attached 1.5 × 1.5 cm slip-resistant tape [3 M Safety-Walk] (used to grip tendon) was sterilized in 70% ethanol and placed on top of the tendon end. The magnets sandwiched the tendon onto the petri dish restraining the fascicle end in place (Fig. 1). The other, free tendon fascicle end was pulled over the third magnet (magnet 3) at the opposing side of the petri dish. A fourth pre-sterilized magnet (magnet 4) was used to hold the second fascicle end in place. Tendons were restrained and placed in a CO_2_ incubator at 37 ^o^C. To harvest mechanically restrained tendons, a sharp blade was used to cut the middle portions of the restrained tendons avoiding any tissues that were directly in contact with the restraining magnets.

### Gene expression

After one day of culture, tendons were submerged in TRIzol to isolate RNA. RNA extraction and relative RT-PCR quantification were performed as previously described with slight modifications [27, 28]. Briefly, Tendon fascicles were homogenized by manually grinding tendons in TRIzol using a Pellet Pestle. Chloroform was then used for phase separation, and a RNA Clean Concentrator Kit (RNA Clean & Concentrator-5; Zymo, Irvine, CA) was used to selectively recover RNA. The RNA was reverse transcribed to cDNA using UltraScript 2.0 cDNA Synthesis Kit (PCR Biosystems, Wayne, PA). 20ng cDNA was used for each PCR reaction. Real-time RT-PCR was performed using qPCRBio Sygreen Blue Mix (PCR Biosystems, Wayne, PA) according to manufacturer’s directions on a Cielo3 real-time PCR system (Azure; Houston, TX, USA). The oligos used in real-time RT-PCR are listed on Table 1. Following PCR reactions, melt curve analysis was performed to validate gene product specificity, respectively. 18 S was selected as the reference gene as we determined there was no difference in the Ct values (average±SE) between floating (9.92±0.51) and restrained (10.06±0.30) conditions (p = 0.81). The mRNA expression levels were derived using deltaCt according to the Pfaffl method [26].

**Table 1:** Oligonucleotide primers used in RT-qPCR.

Gene	Forward primer	Reverse primer	Product size (bp)	Accession no.
18s	GCAATTATTCCCCATGAACG	GGCCTCACTAAACCATCCAA	123	NR_003278.3
Col1	AGCACGTCTGGTTTGGAGAG	GACATTAGGCGCAGGAAGGGT	112	NM_007742.4
Col3	ATCAGGCCAGTGGCAATGTA	CCTTCAGCCTTGAATTCGCC	73	NM_009930.2
Tnc	TGGAATTGCTCCCAGCATCC	CCGGTTCAGCTTCTGTGGTAG	65	NM_011607.3
Acan	GAAAACTTCGGGGTGGG	TCCCTCTGTGACATTACGGG	96	NM_007424.3
Mmp-3	GGTGACCCCACTCACTTTCT	GGCATGAGCCAAGACTGTTC	124	NM_010809.2
Mmp-13	AGACCCCAACCCTAAGCATC	ATAGGGCTGGGTCACACTTC	53	NM_008607.2

### Confocal microscopy

Tissue samples were﻿ fixed in 4% paraformaldehyde/phosphate buffer saline at 4 ^o^C. After 2 h, tissues were washed three times and then immersed in permeabilization/blocking buffer (PBS containing 0.3%Triton, 0.3% bovine serum albumin, and 3% goat serum) at room temperature. After 2 h in permeabilization/blocking buffer, tendons were transferred permeabilization/blocking buffer containing Hoecsht 33342 (1:500), and rhodamine-phalloidin (1:20) (Biotium).

Images were acquired on a Zeiss LSM880 laser-scanning confocal fluorescence microscope (Zeiss, Jena, Germany) with a 20 × 0.8NA objective. Z-stacks were collected using a 0.5 μm step size. Images were processed using Zen software (Zeiss). F-actin intensity was calculated using FIJI software on maximum intensity projection images. Briefly, a rectangular region of interest (ROI) was selected in the middle portion of tendons and the integrated density in the ROI was determined. The average F-actin intensity of the stress deprived controls were set at 100% for each experiment and restrained tendon F-actin values were expressed as a percent of controls. Data was pooled between sets of experiments. To evaluate nuclear morphology, the nuclei from single optical section in the middle regions of z-stacks were outlined using the free-hand tool. The middle regions, which were identified as the region where the nuclei of each cell were the largest in area, were determined manually. Nuclear area and circularity were then calculated.

### Data analysis

Statistical analysis was performed on GraphPad Prism 9. T-tests were used to calculate statistical differences between the two groups of data. P-values less than 0.05 were regarded as statistically significant. Minimum of 3 individual animals per group were used for each experiment. ROUT method was used to determine outliers; however, no outliers were identified in the data sets.

## Results

**Fig. 2:**
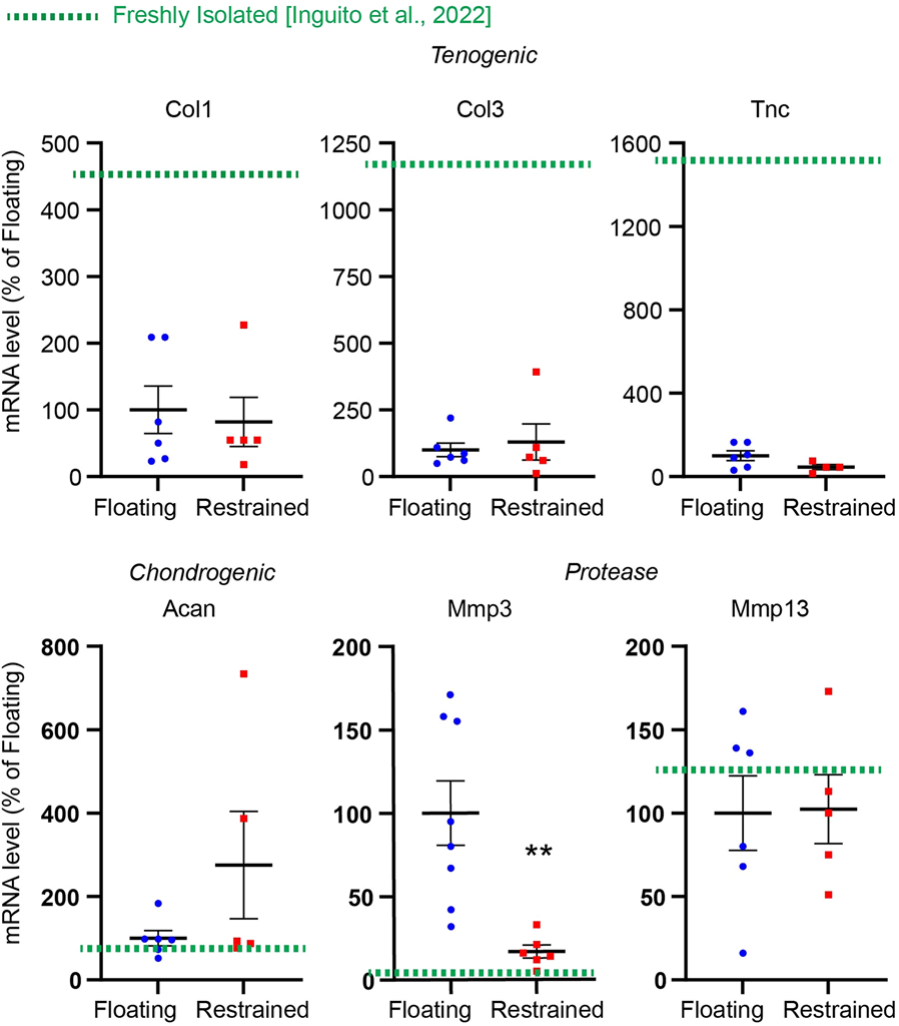
The effect of restraining tendons on tenogenic, chondrogenic, and protease gene expression after 1 day. Dashed lines represent mRNA levels of freshly isolated tendons based off data from Inguito et al. [27]. Mean ± SE; **, p < 0.01 as compared to stress deprived (Floating) control. Each data point in the graph represents biological replicate from one animal

### Restraining tendons decreases Mmp-3 mRNA levels

To determine if restraining tendons represses the effects of stress deprivation, we restrained tendons onto petri dishes using industrial ceramic magnets (Fig. 1). This allowed us to immobilize tendons onto petri dishes and maintained them in a mechanically loaded state.

In a previous study we determined that as compared to freshly isolated tendons, stress deprivation decreases tendon matrix molecules Col1, Col3, Tnc expression [26] (Fig. 2;﻿ Freshly isolated mRNA levels are represented as green, dashed line). While Acan and Mmp13 mRNA levels were not significantly affected, we found stress deprivation upregulates Mmp3. In the present study, we determined that restraining tendons represses Mmp3 mRNA levels 5.9-fold after 1 day of restraining. The expression of Col1, Col3, Tnc, Acan, Mmp-13 remain unchanged (p > 0.05).

### Restraining tendons promotes F-actin and elongated nuclear

**Fig. 3:**
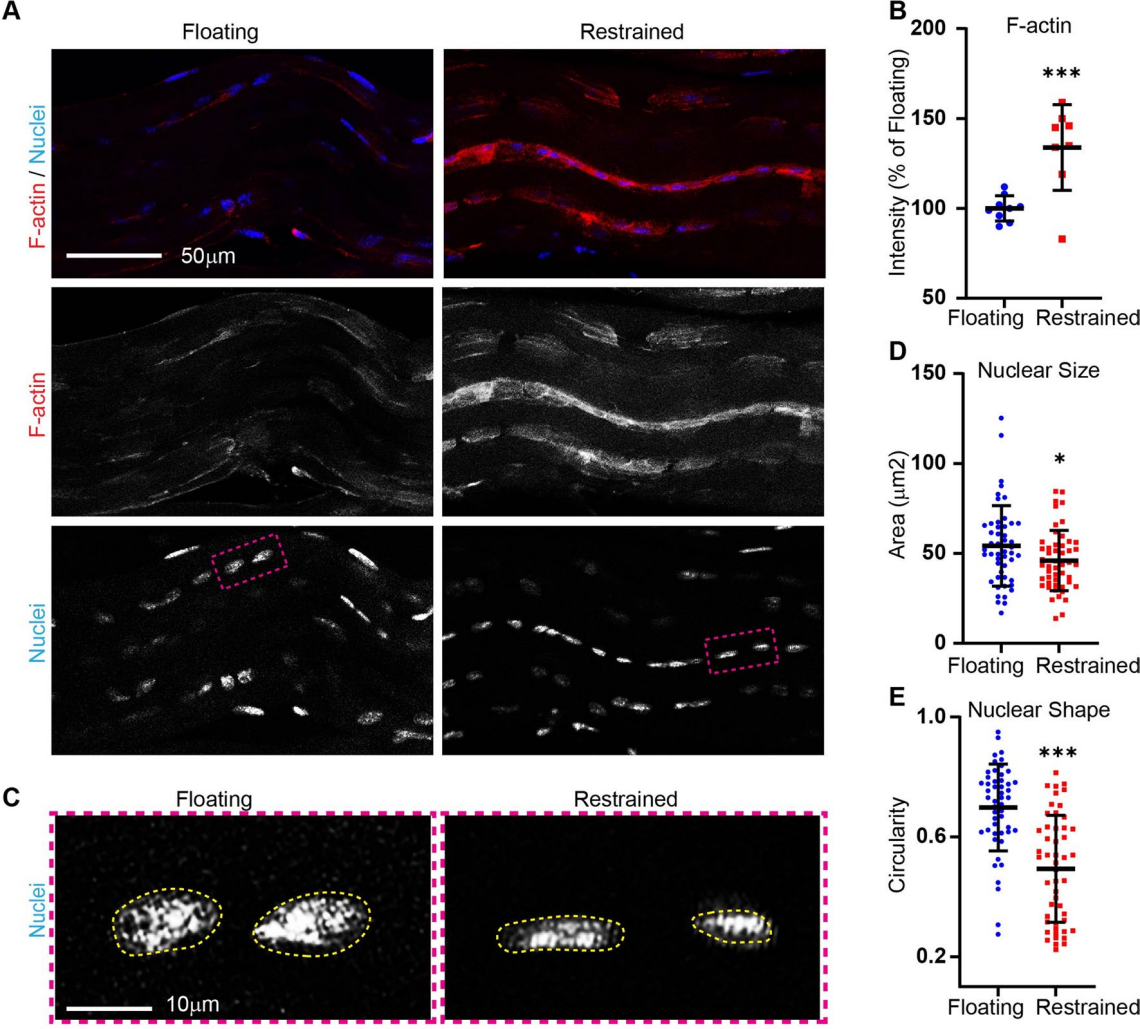
The effect of restraining tendon on F-actin staining and nuclear morphology after 1 day. **A**, **B** Tendons that were mechanically loaded (Restrained) had greater staining for F-actin than stress deprived (Floating) controls. **A**, **C** Nuclei were smaller in area and elongated in the mechanically loaded (Restrained) condition as compared to tendons that were stress deprived in Floating cultures.﻿ **D**, **E**, **F** Quantification of F-actin fluorescent intensity, cell area, and nuclear area. Dot plots demonstrate an overall increase in F-actin staining intensity, and decreases in cell area and circularity, in Restrained as compared to Floating tendons in culture. Mean ± SE; *, p < 0.05 as compared to Floating control; ***, p < 0.001 as compared to Floating control. Each data point represents F-actin intensity/multiple nuclei from individual fascicles. Data from three animals per group were pooled

Previously, we determined that stress deprivation decreases the proportion of F-actin in tendon as compared to freshly isolated tendon. Stretching isolated tenocytes in culture remodels F-actin in order to elicit alterations in gene expression [29]. Therefore, we sought to determine the effect of mechanical restraining on tenocyte F-actin within native tendons. Tendons were stained with phalloidin to visualize F-actin. After 1 day of culture, we determined that restrained tendons have greater staining for F-actin as compared to stress-deprived floating tendons (Fig. 3A, B). Of note, we did not see any evidence of micro-tearing of tendon tissue due to restraining. Furthermore, although strained tendons exhibited crimp (Fig. 3A), the tissues were removed from the mechanical loading system prior to fixation in paraformaldehyde and staining, so the crimp magnitude should not be interpreted as an effect of either floating or being restrained.

Force has been suspected to transmit mechanical load via the F-actin cytoskeleton onto the nucleoskeleton, as reflected by nuclear shape changes, causing structural rearrangement in heterochromatin [30–32]. To determine if nuclear morphology is different between stress deprived and restrained tendons, tenocyte nuclei were stained for Hoechst, and were manually traced (Fig. 3C). Nuclear area (Fig. 3D) and circularity were quantified (Fig. 3E). We determined that nuclear area decreased in restrained tendons. Additionally, we found that restraining tendons decreases nuclear circularity indicating that nuclei are more elongated in restrained versus stress deprived tendons.

## Discussion

In this study we developed a simple model to apply static mechanical loading onto tendon cells in native tendon. Using this new methodology, we demonstrated that restraining tendons preserves nuclear morphology and F-actin organization. This is associated with a specific reduction in Mmp3 levels demonstrating that Mmp3 is a highly mechanosensitive gene.

We developed a simple, cost-effective model that preserves mechanical cellular tension in native mouse tendon tissue, by utilizing commercially available ceramic magnets on standard petri dishes. Therefore, this system has a small footprint and can be readily adapted by other research laboratories. Other simple models such as suspension of weights onto tails exist, however, previous studies use rat tail tendons that are larger and more robust than mouse tendons. Whereas we found that weight suspension on mouse tail tendons to be challenging.

As compared to more complex loading systems, our system has limitations. The amount of load applied to tissues is unknown. Additionally, our system only applies static loading unlike other studies that use bioreactors capable of applying cyclical loading onto tendon tissues. However, our results are consistent with other studies that have applied low magnitude cyclic loading to mouse tendon fascicles. Similar to studies that applied cyclical loading, our static restraining also rescued the detrimental effect of stress deprivation by preserving nuclei morphology and Mmp3 expression compared to native tissue [20]. In addition, like static restraining in the present study, Col1 and Mmp13 were not altered by the application of cyclical load. Albeit cyclical loading did result in additional mRNA level changes to other genes (Ctgf, Scx and Mmp9) which we did not investigate using our system. Of note, the balance between Timps and the Mmps is essential to determine the protection afforded by mechanical loading. To gain insight into this mechanoprotection a ratio of Mmp/Timp should be considered as previously shown by others [33–35]. Nevertheless, this demonstrates that simply restraining tissues provide enough static mechanical load to produce similar gene regulation results as cyclical loading.

Restraining tissues also affects F-actin and nuclear morphology. It has been suggested that F-actin is a hardwire between the extracellular space and the nucleus, and may be responsible for transmitting mechanical forces [21, 33, 36]. Our study demonstrates that as compared to the mechanically loaded condition, stress deprivation via floating cultures reduces F-actin. In turn, this results in altered nuclear morphology. The altered nuclear morphology can change chromatin conformation allowing access to the promoter region of certain genes [37]. We suspect that access to the Mmp3 promoter region is altered, however, this was not tested here and is a matter of future investigation.

Our finding supports that Mmp3 is a highly mechanosensitive gene. Minimal mechanical loading by restraining produces enough tension to suppress Mmp3 expression. Notably, in the present study we focused on alterations in gene expression. While we did not examine MMP3 protein levels or activity, previous studies have demonstrated changes in MMP3 protein by mechanical stretch is correlated with the mRNA level in osteoblast and tendon cells [38, 39]. The regulation of Mmp3 by mechanical load is consistent with studies that examined other cell types. We previously showed that static loading generated by 3D collagen gel contraction reduces Mmp3 expression in osteoblasts as compared to floating collagen gels [15, 16]. Other types of mechanical loading such as shear stress, and compression also change the expression of Mmp3 in bone, cartilage and tendon cells or tissues [11, 12, 19, 40–42]. Tendon cells can experience all types of mechanical loading, loading including, tensile, shear, and compression loads [43] which could lead to regulation of Mmp3. Physiologic level of mechanical stimulation maintains a low expression of Mmp3 in the tendon; while underloading increases the expression [18, 44, 45] which may contribute to matrix degradation [17, 18]. Therefore, the mechanical regulation of Mmp3 appears to play a central role in tendon tissue homeostasis and injury. A further understanding on the mechanoregulation of key tendon matrix genes may enable the development of new strategies to prevent degradation during tendon rupture or detachment.

## Data Availability

The data used and/or analyzed are available from the corresponding author on reasonable request.
